# Establishment of a prognostic signature for lung adenocarcinoma by integration of 7 pyroptosis-related genes and cross-validation between the TCGA and GEO cohorts: A comprehensive bioinformatics analysis

**DOI:** 10.1097/MD.0000000000029710

**Published:** 2022-07-22

**Authors:** Wei Zhang, Shiqian Wan, Zhaohui Qu, Jing Ge, Chunxia Zhang, Chunfang Li, Yingchun Jiang

**Affiliations:** a Department of Critical Care Medicine, Wuhan Jinyintan Hospital, Wuhan, Hubei, People’s Republic of China; b Department of Infectious Diseases, Wuhan Jinyintan Hospital, Wuhan, Hubei, People’s Republic of China; c Department of Ultrasound Diagnostics, Wuhan Jinyintan Hospital, Wuhan, Hubei, People’s Republic of China.

**Keywords:** cox regression analysis, Kaplan-Meier survival analysis, lung adenocarcinoma, prognostic signature, pyroptosis, receiver operating characteristic curve analysis

## Abstract

Pyroptosis-related genes (PRGs) have been reported to be associated with prognosis of lung adenocarcinoma (LUAD). Until now, the relationship of PRGs to the prognosis of LUAD patients and its underlying mechanisms have been poorly elucidated. Using The Cancer Genome Atlas (TCGA) LUAD cohort, a prior bioinformatics analysis constructed a prognostic signature incorporating 5 PRGs (*NLRP7*, *NLRP1*, *NLRP2*, *NOD1*, and *CASP6*) for predicting prognosis of LUAD patients. However, it has not been validated by the Gene Expression Omnibus (GEO) LUAD cohort yet. We implemented a modified bioinformatics analysis to, respectively, construct one prognostic signature with the TCGA cohort and with the GEO cohort and attempted to perform cross-validations by the GEO cohort and the TCGA cohort alternately in turn. Univariate and multivariate Cox regression analysis screened PRGs and constructed 2 prognostic signatures with the TCGA and GEO cohorts. All LUAD samples were classified into high- and low-risk groups according to the median risk score that was generated by regression formula. Kaplan-Meier survival analysis compared the overall survival rate between the 2 risk groups, and receiver operating characteristic curve analysis evaluated predictive performance of the 2 signatures. Additionally, risk score, combined with clinicopathological features, was subjected to multivariate Cox regression analysis, to evaluate independent prognostic value of the 2 signatures. Finally, the 2 signatures received cross-validations by the GEO and TCGA cohorts, alternately. The TCGA cohort yielded a 3-gene signature (*PYCARD*, *NLRP1*, and *NLRC4*), whereas the GEO cohort built a 7-gene signature (*SCAF11*, *NOD1*, *NLRP2*, *NLRP1*, *GPX4*, *CASP8*, and *AIM2*) for predicting the prognosis of LUAD patients. Multivariate analysis proved independent prognostic value of risk score in the TCGA cohort (hazard ratio, = 1.939,; *P* = 8.43 × 10^−4^) and the GEO cohort (hazard ratio, = 2.291,; *P* = 4.34 × 10^−9^). Cross-validations confirmed prognostic value for the 7-gene signature from the GEO cohort by the TCGA cohort but not for the 3-gene signature from the TCGA cohort by the GEO cohort. We develop and validate a 7-gene prognostic signature (*SCAF11*, *NOD1*, *NLRP2*, *NLRP1*, *GPX4*, *CASP8*, and *AIM2*) with independent prognostic value for patients with LUAD.

## 1. Introduction

Lung cancer is a fatal malignancy worldwide and is one of the leading etiologies of death caused by malignant tumors, with a poor 5-year survival rate of around 15%.^[1]^ About 60% of lung cancer cases are diagnosed with histological adenocarcinoma.^[2]^ Reliable novel therapeutic targets and prognostic signatures are urgently needed to make targeted therapies more feasible and to achieve a better prognosis for lung cancer, especially for lung adenocarcinoma (LUAD).

Pyroptosis, also known as cellular inflammatory necrosis,^[3]^ is a novel form of programmed cell death.^[4]^ It is characterized by cellular swelling with balloon-like bubbling,^[5]^ release of inflammatory cell cytokines,^[6]^ and leakage of lactate dehydrogenase in the plasma membrane.^[7]^ When pyroptosis occurs, a variety of danger-associated signaling molecules and cytokines are activated and released, accompanied by a strong inflammatory response and activation of the immune system.^[8]^ Until now, the relationship of pyroptosis-related genes (PRGs) to the prognosis of LUAD patients and its underlying mechanisms have been poorly elucidated.

Recently, Lin et al^[9]^ published a systematic bioinformatics analysis, identifying a prognostic signature that contained 5 PRGs (NLR family pyrin domain containing [*NLRP*] *7*, *NLRP1*, *NLRP2*, nucleotide binding oligomerization domain [*NOD*] *1*, and cysteine-aspartic acid protease [*CASP*] 6) for predicting the prognosis of patients with LUAD. However, they confessed a limitation that all their analyses were conducted using The Cancer Genome Atlas (TCGA) LUAD cohort but were not validated using the Gene Expression Omnibus (GEO) LUAD cohort. Besides, there existed a relative lack and imbalance of the data for normal lung tissues from the TCGA cohort, compared to that for tumor tissues (normal, 59; tumor, 526), which was likely to introduce some bias to their results concerning the identification of differentially expressed genes (DEGs) and hub genes.

Therefore, we implemented a modified bioinformatics analysis to, respectively, construct one prognostic signature with the TCGA cohort and the GEO cohort and attempted to perform cross-validations by the GEO cohort and the TCGA cohort alternately in turn.

## 2. Materials and Methods

### 2.1. Ethical approval

This study was approved by the Ethics Committee of Wuhan Jinyintan Hospital (No. KY-2021-06.01).

### 2.2. Identification of DEGs

In order to identify the DEGs between normal lung and tumor tissues, we downloaded the RNA sequencing data and the corresponding clinicopathological information of 585 samples (normal, 59; tumor, 526) that were deposited in the LUAD cohort of the TCGA database on June 21, 2021 (https://portal.gdc.cancer.gov/repository). Due to a deficiency of data from normal lung tissues in the TCGA cohort, we also extracted data of 288 normal lung samples from the Genotype-Tissue Expression Project (GTEx) database (https://xenabrowser.net/datapages/). Accordingly, the expression and clinicopathological data from a total of 873 samples (normal, 347; tumor, 526) were obtained. The expression data were normalized to fragments per kilobase million values before comparison. The “limma” R package was used to screen and identify DEGs. We referred 33 genes from prior reviews,^[6,10–12]^ which have been well acknowledged as PRGs and are listed in Table 1. Since there were 3 of the 33 genes, *NLRP2*, *NLRP6*, and *NLRP7*, whose expression data were not available in the TCGA database, we had to use the expression levels of the remaining 30 for comparison and identification. To examine the intercommunication of these PRGs, we undertook a protein-protein interaction (PPI) analysis. A PPI network for these differentially expressed PRGs was constituted with Search Tool for the Retrieval of Interacting Genes, version 11.0 (https://string-db.org/).

**Table 1:** Thirty-three pyroptosis-related genes.

Genes	Full names
*AIM2*	Absent in melanoma 2
*CASP1*	Cysteine-aspartic acid protease-1
*CASP3*	Cysteine-aspartic acid protease-3
*CASP4*	Cysteine-aspartic acid protease-4
*CASP5*	Cysteine-aspartic acid protease-5
*CASP6*	Cysteine-aspartic acid protease-6
*CASP8*	Cysteine-aspartic acid protease-8
*CASP9*	Cysteine-aspartic acid protease-9
*ELANE*	Elastase, neutrophil expressed
*GPX4*	Glutathione peroxidase 4
*GSDMA*	Gasdermin A
*GSDMB*	Gasdermin B
*GSDMC*	Gasdermin C
*GSDMD*	Gasdermin D
*GSDME*	Gasdermin E
*IL18*	Interleukin 18
*IL1B*	Interleukin 1 beta
*IL6*	Interleukin 6
*NLRC4*	NLR family CARD domain containing 4
*NLRP1*	NLR family pyrin domain containing 1
*NLRP2*	NLR family pyrin domain containing 2
*NLRP3*	NLR family pyrin domain containing 3
*NLRP6*	NLR family pyrin domain containing 6
*NLRP7*	NLR family pyrin domain containing 7
*NOD1*	Nucleotide binding oligomerization domain containing 1
*NOD2*	Nucleotide binding oligomerization domain containing 2
*PJVK*	Pejvakin/deafness, autosomal recessive 59
*PLCG1*	Phospholipase C gamma 1
*PRKACA*	Protein kinase cAMP-activated catalytic subunit alpha
*PYCARD*	PYD and CARD domain containing
*SCAF11*	SR-related CTD associated factor 11
*TIRAP*	TIR domain containing adaptor protein
*TNF*	Tumor necrosis factor

### 2.3. Tumor classification by consensus clustering analysis

We made a consensus clustering analysis of all the 526 LUAD samples from the TCGA cohort by the “ConsensusClusterPlus” R package to figure out the connections between the expression of the 29 differentially expressed PRGs and LUAD subtypes, referring to Ye et al’s^[13]^ and Wang et al’s^[14]^ methodology.

### 2.4. Functional enrichment analysis

To unveil the differences in the enrichment activity of gene functions and pathways between normal lung and tumor tissues, we carried out gene ontology (GO) enrichment analysis and Kyoto Encyclopedia of Genes and Genomes (KEGG) pathway analysis based on the 29 differentially expressed PRGs by applying the “clusterProfiler,” “org.Hs.eg.db,” “enrichplot,” and “ggplot2” R packages.^[9,13,14]^

### 2.5. Development of prognostic signature

A total of 513 samples in the TCGA cohort and 443 in the GEO cohort were, respectively, matched with corresponding patients who had complete survival information. A univariate Cox regression analysis was fulfilled for preliminary screening of the survival-related genes in the TCGA and GEO cohorts. To avoid omissions, we set 0.2 as the threshold *P* value.^[13,15]^ The least absolute shrinkage and selection operator regression analysis by the “glmnet” R package was utilized to narrow down the number of candidate genes for multivariate Cox regression analysis. Then, the multivariate Cox regression analysis in the TCGA and GEO cohorts was undertaken to construct prognostic signature and to determine regression coefficient for each gene that was incorporated in the prognostic signatures. Also, a risk score of overall mortality for each LUAD patient was generated according to the multivariate Cox regression formula in the TCGA and GEO cohorts. Hence, all the samples in the TCGA and GEO cohorts were, respectively, allocated into high- and low-risk groups according to median values of risk scores. The principal component analysis (PCA) based on the prognostic signatures was performed by the “prcomp” function in the “stats” R package. The overall survival (OS) time and OS rate of LUAD patients was compared between high- and low-risk groups by the Kaplan-Meier survival analysis and 2-sided log-rank test in the TCGA and GEO cohorts. In order to evaluate the prediction performance of the prognostic signatures, a 3-year receiver operating characteristic (ROC) curve analysis in the TCGA and GEO cohorts was, respectively, done using the “survival,” “survminer,” and “timeROC” R packages.^[9,13,14]^

### 2.6. Evaluation of prognostic value of risk score

We retrieved the clinicopathological information including age, gender, tumor stage (stage), the size and extent of the main tumor (T), regional lymph node (N) and metastasis (M), and grade for tumor differentiation (grade) of patients in the TCGA and GEO cohorts. These variables were analyzed in conjunction with the risk scores that were yielded by our prognostic signatures. Univariate and multivariable Cox regression analyses were conducted to testify whether the risk scores were independent prognostic indicators for predicting risk of overall mortality among LUAD patients in the TCGA and GEO cohorts.^[9,13,14]^

### 2.7. Cross-validations of prognostic signatures

In order to appraise the robustness of the 2 prognostic signatures that were, respectively, constructed with the TCGA and GEO cohorts, we in turn attempted validation of the 2 prognostic signatures by the GEO and TCGA cohorts alternately. By utilizing the median risk scores, the patients in the GEO and TCGA cohorts were also categorized into low- or high-risk groups. The Kaplan-Meier survival analysis and time-dependent ROC curve analysis were also undertaken to compare the OS rate and time between the 2 risk groups and to, respectively, evaluate the prognostic value of the 2 signatures in the GEO and TCGA cohorts.

### 2.8. Comparison of immune activity between 2 risk groups

We compared the enrichment scores of 16 types of immune cells and the activity of 13 immune-related pathways between low- and high-risk groups in both the TCGA and GEO cohorts by single-sample gene set enrichment analysis (ssGSEA), applying the “GSVA,” “GSEABase,” “ggplot2,” “ggpubr,” and “reshape2” R packages.^[9,13,14]^

### 2.9. Statistical analysis

All statistical analyses were accomplished using the R Software, version 4.0.5. Continuous variables were expressed as median (interquartile range) and compared using the Mann-Whitney *U* test, while categorical variables are expressed as frequencies (n [%]) and compared using the χ^2^ or Fisher exact test, when appropriate. All analyses were 2 tailed, and a *P* value of <.05 was considered statistically significant.

## 3. Results

### 3.1. DEGs and hub genes

**Figure 1.**
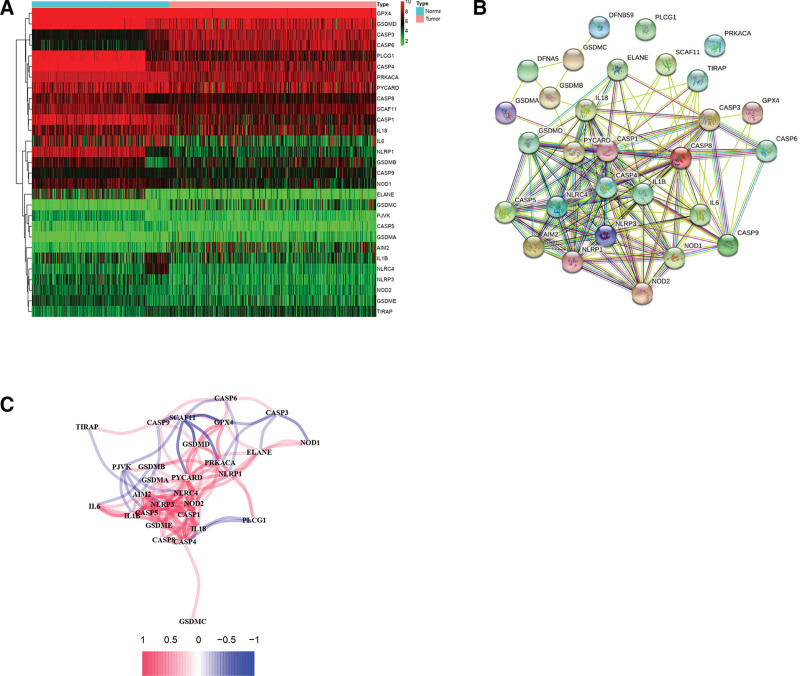
Expression profile of and interactions across 29 pyroptosis-related DEGs. (A) Heat map for expression levels of 29 pyroptosis-related DEGs (red: higher gene expression; green: lower gene expression). (B) Protein-protein interaction network showing interactions across 29 pyroptosis-related DEGs (minimum interaction score, 0.9). (C) Correlation network for 29 pyroptosis-related DEGs (red line: positive correlation; blue line: negative correlation. Color depth reflects strength of relevance). DEG = differentially expressed gene.

After comparing expression levels of 30 PRGs between normal lung and tumor tissues in the TCGA cohort, we singled out 29 DEGs (all *P* < 0.05; Table 2). Among them, 22 genes (*NLRP1*, interleukin [*IL*]-6, *PLCG1*, *ELANE*, *CASP1*, *CASP4*, *GSDMD*, *NOD1*, *NLRP3*, *PRKACA*, *IL1B*, *CASP8*, *NLRC4*, *NOD2*, *PJVK*, *IL18*, *GSDME*, *GSDMB*, *SCAF11*, *CASP5*, *PYCARD*, and *CASP9*) were downregulated while the other seven (glutathione peroxidase 4 [*GPX4*], *TIRAP*, *GSDMA*, *GSDMC*, absent in melanoma 2 [*AIM2*], *CASP3*, and *CASP6*) were upregulated. The expression levels of these 29 DEGs are displayed as a heat map in Figure 1A. The PPI across the DEGs is visualized in Figure 1B. The top 10 hub genes, *CASP8*, *IL18*, *PYCARD*, *CASP1*, *IL1B*, *IL6*, *NLRP1*, *NLRC4*, *NLRP3*, and *CASP4*, were determined by intersection of 12 topological algorithms of CytoHubba plugin in the Cytoscape software. The correlation network depicting all the 29 pyroptosis-related DEGs is presented in Figure 1C.

**Table 2:** Expression levels of 30 pyroptosis-related genes between normal lung and tumor tissues in the The Cancer Genome Atlas cohort.

Gene	Normal mean	Tumor mean	LogFC	*P* value	FDR
*TNF*	1.128	1.137	0.009	.515	0.520
*GPX4*	7.108	7.202	0.094	.004	0.005
*PYCARD*	4.158	3.861	−0.297	2.59 × 10^−5^	2.93 × 10^−5^
*IL1B*	2.286	1.847	−0.439	2.39 × 10^−6^	2.76 × 10^−6^
*IL18*	3.711	3.362	−0.350	1.72 × 10^−8^	2.06 × 10^−8^
*GSDMB*	2.962	2.621	−0.341	3.87 × 10^−10^	4.74 × 10^−10^
*NLRC4*	1.633	1.259	−0.374	8.78 × 10^−12^	1.10 × 10^−11^
*CASP9*	2.666	2.482	−0.184	1.29 × 10^−12^	1.65 × 10^−12^
*GSDME*	1.826	1.483	−0.343	6.76 × 10^−18^	9.27 × 10^−18^
*CASP8*	3.508	3.113	−0.395	1.40 × 10^−23^	2.07 × 10^−23^
*NOD2*	1.712	1.338	−0.374	1.36 × 10^−25^	2.06 × 10^−25^
*TIRAP*	1.560	1.929	0.369	1.46 × 10^−33^	2.43 × 10^−33^
*CASP5*	0.781	0.450	−0.332	3.17 × 10^−35^	5.39 × 10^−35^
*GSDMA*	0.338	0.757	0.419	2.14 × 10^−35^	3.63 × 10^−35^
*PJVK*	1.130	0.767	−0.363	9.90 × 10^−37^	1.72 × 10^−36^
*NLRP3*	1.895	1.196	−0.699	1.27 × 10^−41^	2.37 × 10^−41^
*SCAF11*	3.838	3.503	−0.334	7.32 × 10^−43^	1.38 × 10^−42^
*IL6*	3.855	2.013	−1.842	1.20 × 10^−47^	2.42 × 10^−47^
*AIM2*	0.873	2.213	1.340	3.71 × 10^−55^	8.28 × 10^−55^
*NOD1*	3.277	2.454	−0.823	9.44 × 10^−71^	2.80 × 10^−70^
*GSDMD*	5.413	4.402	−1.011	1.76 × 10^−72^	5.43 × 10^−72^
*GSDMC*	0.422	1.657	1.234	2.17 × 10^−75^	7.12 × 10^−75^
*PRKACA*	4.477	3.926	−0.551	3.59 × 10^−88^	1.70 × 10^−87^
*CASP4*	5.219	3.918	−1.301	1.96 × 10^−89^	9.62 × 10^−89^
*PLCG1*	5.132	3.398	−1.734	1.58 × 10^−89^	7.82 × 10^−89^
*ELANE*	2.020	0.431	−1.590	1.86 × 10^−90^	9.45 × 10^−90^
*CASP1*	4.571	3.148	−1.423	6.57 × 10^−100^	5.05 × 10^−99^
*NLRP1*	4.014	1.940	−2.074	1.05 × 10^−112^	1.65 × 10^−111^
*CASP6*	2.553	4.111	1.558	3.26 × 10^−126^	2.21 × 10^−124^
*CASP3*	2.786	4.196	1.410	2.01 × 10^−127^	1.60 × 10^−125^

Normal mean: mean value of gene expression level in normal lung tissues; tumor mean: mean value of gene expression level in tumor tissues.

FDR = false discovery rate; logFC = logarithmic fold change of gene expression level.

### 3.2. Tumor classification

**Figure 2.**
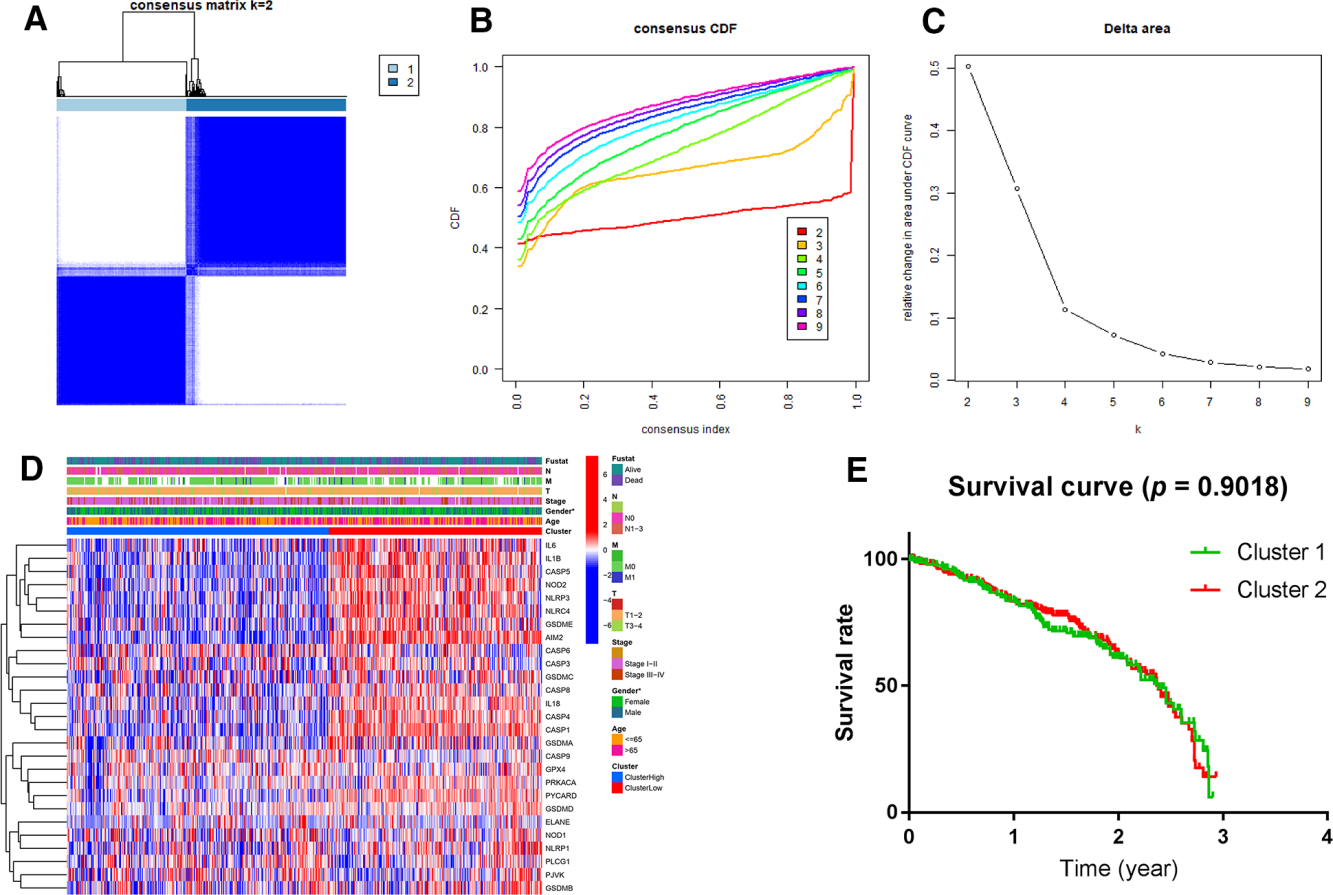
Tumor classification by CCA based on 29 pyroptosis-related DEGs. (A–C) CCA of 29 pyroptosis-related DEGs, inferring optimal number of clusters, the lowest proportion of ambiguous clustering and best CDF value by taking the *k* value of 2. According to the consensus clustering matrix (*k* = 2), 526 lung adenocarcinoma samples were grouped into 2 clusters. (D) Heat map and clinicopathologic characters of the 2 clusters classified by CCA (Fustat: survival status; T: stage for tumor; N: stage for regional lymph node; M: stage for metastasis). (E) Kaplan-Meier curves for comparison of overall survival rate between the 2 clusters classified by CCA. CCA = consensus clustering analysis, CDF = cumulative distribution function, DEG = differentially expressed gene.

By escalating the clustering parameter (*k*) from 2 to 10 in consensus clustering analysis, we discerned that when *k* = 2, the intragroup correlations were the highest and the intergroup correlations were the lowest, denoting that the 526 LUAD samples could be well separated into 2 clusters according to the 29 pyroptosis-related DEGs (Fig. 2A–2C). The gene expression profile and the clinicopathological attributes including cluster (cluster 1 or 2), gender (female or male), age (≤65 or >65 years), survival status (alive or dead), N (N0 or N1–3), T (T1–2 or T3–4), M (M0 or M1), and stage (stage I–II or stage III–IV) are exhibited in a heat map (Fig. 2D). The OS rate was compared between the 2 clusters, but no apparent diversity was detected (*P* = 0.902; Fig. 2E).

### 3.3. Functional enrichment

**Figure 3.**
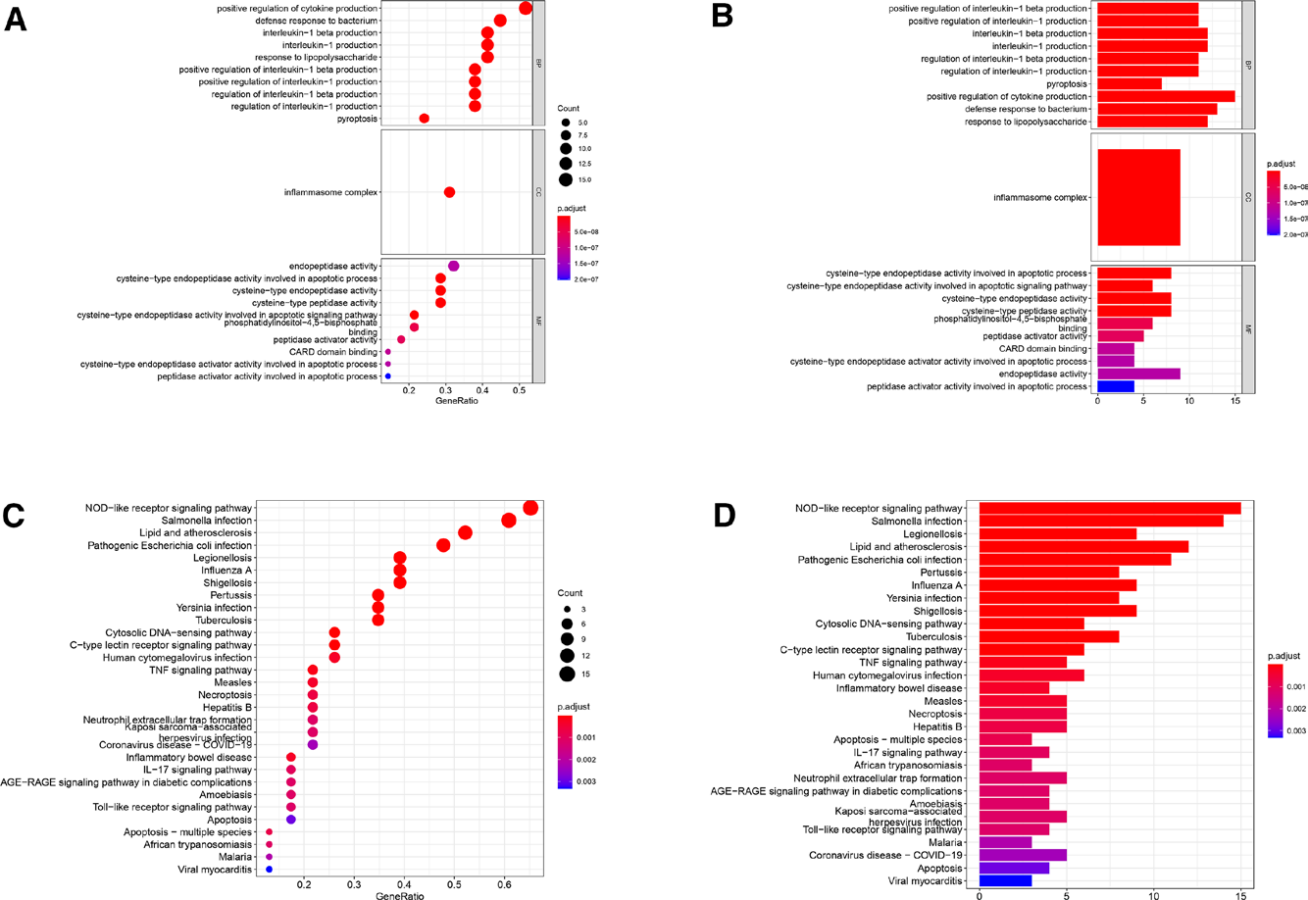
GO enrichment analysis and KEGG pathway analysis based on pyroptosis-related DEGs with pooled Genotype-Tissue Expression Project and The Cancer Genome Atlas cohort. (A) Bubble chart for GO enrichment of pyroptosis-related DEGs. Size of bubble indicates the number of significant genes in the given enriched term. Color denotes adjusted *P* value. The closer the color is to red, the more significant the enrichment is. (B) Barplot chart for GO enrichment of pyroptosis-related DEGs. Length of the bar indicates the number of significant genes in the given enriched term. Color signifies adjusted *P* value. The closer the color is to red, the more significant the enrichment is. (C) Bubble chart for KEGG pathway of pyroptosis-related DEGs. (D) Barplot chart for KEGG pathways of pyroptosis-related DEGs. COVID-19 = coronavirus disease 2019, DEG = differentially expressed gene, GO = gene ontology, KEGG = Kyoto Encyclopedia of Genes and Genomes, IL = interleukin, NOD = nucleotide binding oligomerization domain, TNF = tumor necrosis factor.

The GO enrichment analysis and KEGG pathway analysis were then conducted based on these DEGs. The GO enrichment analyses manifested that DEGs in terms of biological processes were mainly enriched in the production and regulation of cytokines (IL-1 and IL-1B) and pyroptosis; that of cellular components were mostly enriched in the inflammasome complex; that of molecular function were primarily enriched in the endopeptidase activity, cysteine-type peptidase activity, and cytokine receptor binding (Fig. 3A and 3B). The KEGG pathway analyses demonstrated that DEGs were involved in dysregulated pathway of NOD-like receptor signaling, cytosolic DNA sensing, tumor necrosis factor signaling, necroptosis, neutrophil extracellular trap formation, Toll-like receptor signaling, apoptosis, p53 signaling, and nuclear factor (NF)-kappa B signaling (Fig. 3C and 3D).

### 3.4. Development of prognostic signature with the TCGA cohort

**Figure 4.**
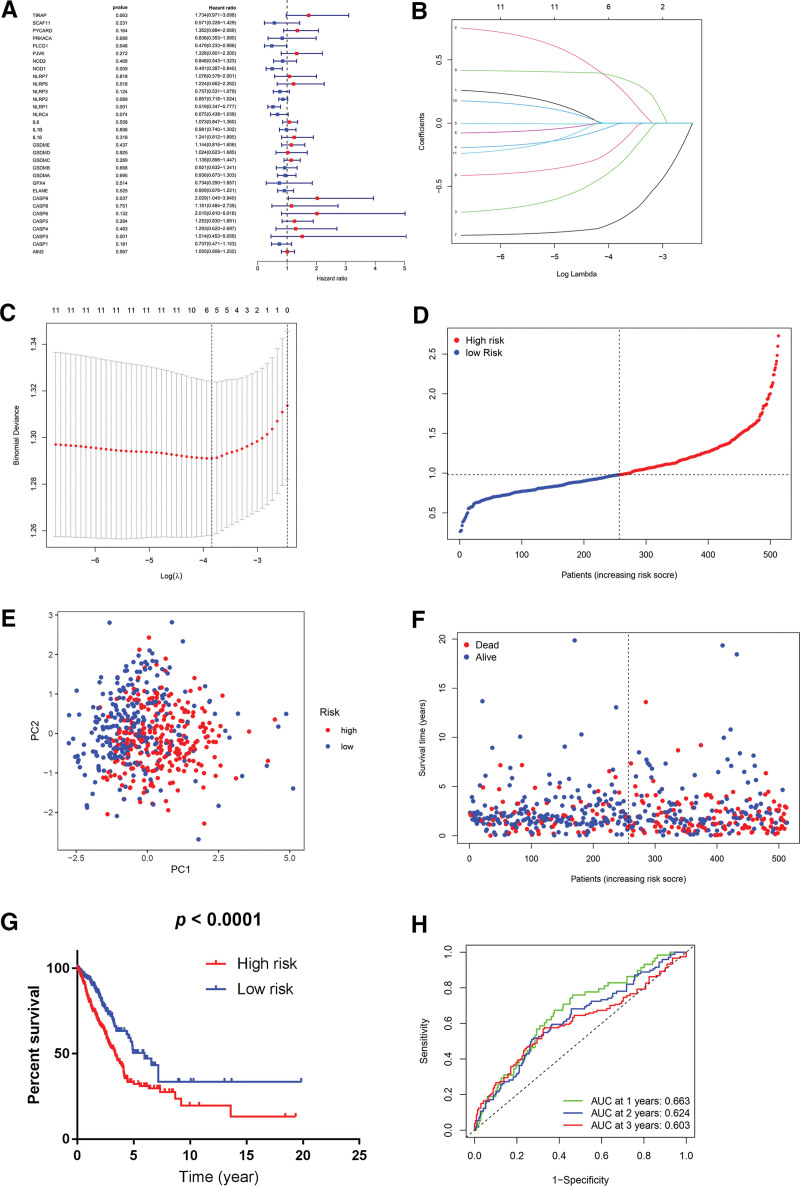
Construction of prognostic signature with the TCGA cohort. (A) Univariate Cox regression analysis of OS for 32 pyroptosis-related genes. (B) LASSO regression analysis of 11 candidate prognosis-related genes for multivariate Cox regression analysis. (C) Cross-validation for tuning the parameter selection in the LASSO regression analysis. (D) Distribution of the number of patients in low- and high-risk groups based on median value of risk scores. (E) Principal component analysis plot based on risk scores, separating LUAD patients into 2 clusters. (F) Survival status for each patient in the TCGA cohort (low-risk group: on the left side of the dotted line; high-risk population: on the right side of the dotted line; orange: higher risk score; blue: lower risk score). (G) Kaplan-Meier survival curves for OS of LUAD patients in the high- and low-risk groups, presenting a lower OS rate in the high-risk group than in the low-risk group. (H) Receiver operating characteristic curves of predictive performance of prognostic signature for LUAD patients. AUC = area under the curve, LASSO = least absolute shrinkage and selection operator, LUAD = lung adenocarcinoma, OS = overall survival, PC = principal component, TCGA = The Cancer Genome Atlas.

After univariate Cox regression analysis, there were 11 genes (*PYCARD*, *GSDMC*, *CASP6*, *NLRP3*, *CASP1*, *TIRAP*, *NLRC4*, *PLCG1*, *CASP9*, *NOD1*, and *NLRP1*) that met the criteria of *P* < .2^[13,15]^ (Fig. 4A) and were preserved for least absolute shrinkage and selection operator regression analysis, which narrowed down the number of candidate genes for multivariate Cox regression analysis to six (*PYCARD*, *PLCG1*, *NOD1*, *NLRP1*, *NLRC4*, and *CASP9*; Fig. 4B and 4C). After multivariate Cox regression analysis was undertaken, 3 genes (*PYCARD*, *NLRP1*, and *NLRC4*), together with their regression coefficients, were accepted for establishing a prognostic signature (Table 3). The risk scores for overall mortality of LUAD patients in the TCGA cohort were calculated by using the following formula: risk score = (0.648 × PYCARD exp.) + (−0.770 × NLRP1 exp.) + (−0.383 × NLRC4 exp.). Then, the 513 samples were classified into high- and low-risk groups according to the median value of risk scores (Fig. 4D). The PCA uncovered that patients with different risks were clearly separated into 2 clusters (Fig. 4E). The patients in the high-risk group had more deaths and a shorter survival time than those in the low-risk group (Fig. 4F). The Kaplan-Meier survival analysis and log-rank test indicated a notably longer OS time (5.96 vs 3.37 years; *P* < 0.0001) and a higher lower 5-year OS rate (29% vs 24%; *P* < 0.001) in the low-risk group than in the high-risk group (Fig. 4G). Time-dependent ROC curve analysis for the 3-gene prognostic signature demonstrates that the area under the curve (AUC) value was 0.663 for 1-year, 0.624 for 2-year, and 0.603 for 3-year survival (Fig. 4H).

**Table 3:** Multivariate Cox regression analysis of candidate genes for construction of prognostic signature.

Cohort	Gene	Coef.	HR	95% CI		*P* value
				LL	UL	
TCGA	*PYCARD*	0.648	1.911	1.203	3.037	.006
	*NLRP1*	−0.770	0.463	0.299	0.717	<.001
	*NLRC4*	−0.383	0.682	0.428	1.085	.106
GEO	*SCAF11*	−0.280	0.756	0.557	1.025	.071
	*NOD1*	−0.261	0.770	0.565	1.049	.098
	*NLRP2*	−0.076	0.927	0.854	1.006	.070
	*NLRP1*	−0.141	0.868	0.729	1.035	.115
	*GPX4*	−0.301	0.740	0.557	0.982	.037
	*CASP8*	0.293	1.341	1.029	1.747	.030
	*AIM2*	0.131	1.141	1.010	1.289	.035

CI = confidence interval, Coef. = coefficient, GEO = Gene Expression Omnibus, HR = hazard ratio, LL = lower limit, TCGA = The Cancer Genome Atlas, UL = upper limit.

### 3.5. Development of prognostic signature with the GEO cohort

**Figure 5.**
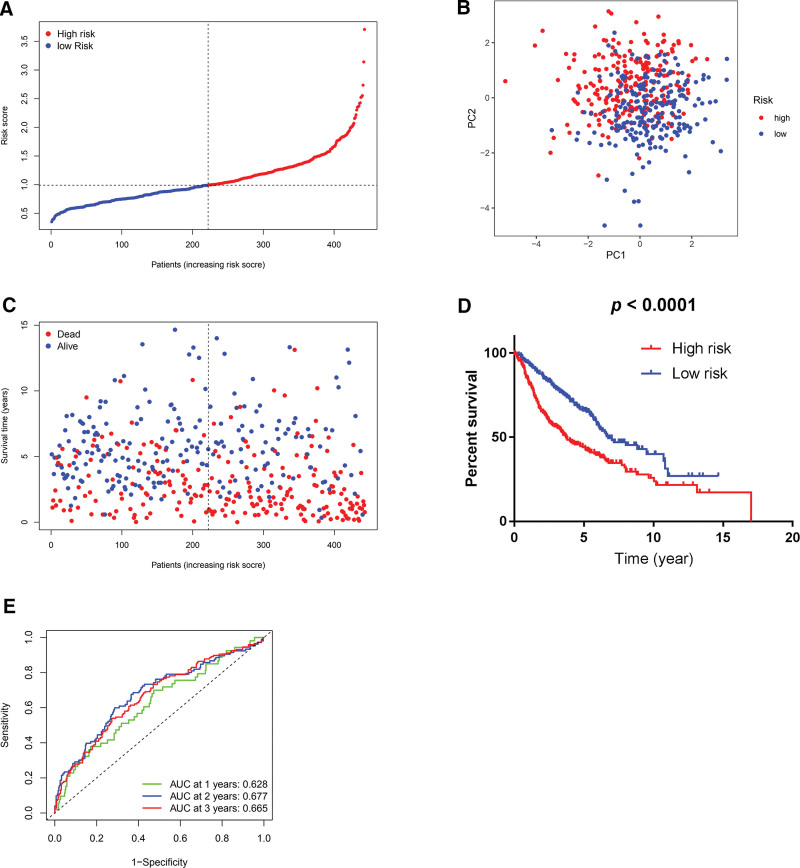
Construction of prognostic signature with the GEO cohort. (A) Distribution of the number of patients in low- and high-risk groups based on the median value of risk scores. (B) Principal component analysis plot based on risk scores, separating LUAD patients into 2 clusters. (C) Survival status for each patient in the GEO cohort (low-risk group: on the left side of the dotted line; high-risk population: on the right side of the dotted line; orange: higher risk score; blue: lower risk score). (D) Kaplan-Meier survival curves for OS of LUAD patients in high- and low-risk groups, indicating a lower OS rate in the high-risk group than in the low-risk group. (E) Receiver operating characteristic curves of predictive performance of prognostic signature for LUAD patients. AUC = area under the curve, GEO = Gene Expression Omnibus, LUAD = lung adenocarcinoma, OS = overall survival.

Likewise, a 7-gene signature merging *SCAF11*, *NOD1*, *NLRP2*, *NLRP1*, *GPX4*, *CASP8*, and *AIM2* was formulated with the GEO cohort by multivariate Cox regression analysis (Table 3). The risk scores were computed as follows: risk score = (−0.280 × SCAF11 exp.) + (−0.261 × NOD1 exp.) + (−0.076 × NLRP2 exp.) + (−0.141 × NLRP1 exp.) + (−0.301 × GPX4 exp.) + (0.293 × CASP8 exp.) + (0.131 × AIM2 exp.). Based on the median value of risk scores, 222 patients in the GEO cohort were sorted into the low-risk group, while the other 221 were sorted into the high-risk group (Fig. 5A). The PCA indicated satisfactory separation between the 2 groups (Fig. 5B). The patients in the low-risk group were found to have longer OS times and lower death rates than those in the high-risk group (Fig. 5C). Parallelly, the Kaplan-Meier analysis confirmed the prognostic ability of our 7-gene risk signature by indicating an appreciably higher 5-year survival rate (65.7% vs 44.0%; *P* < 0.0001) and a longer OS time (6.74 vs 3.75 years; *P* < 0.0001) in the low-risk group than in the high-risk group (Fig. 5D). The ROC curve analysis for the GEO cohort also showed moderate predictive performance (AUC, 0.628 for 1-year, 0.677 for 2-year, and 0.665 for 3-year survival; Fig. 5E).

### 3.6. Independent prognostic value of risk score

**Figure 6.**
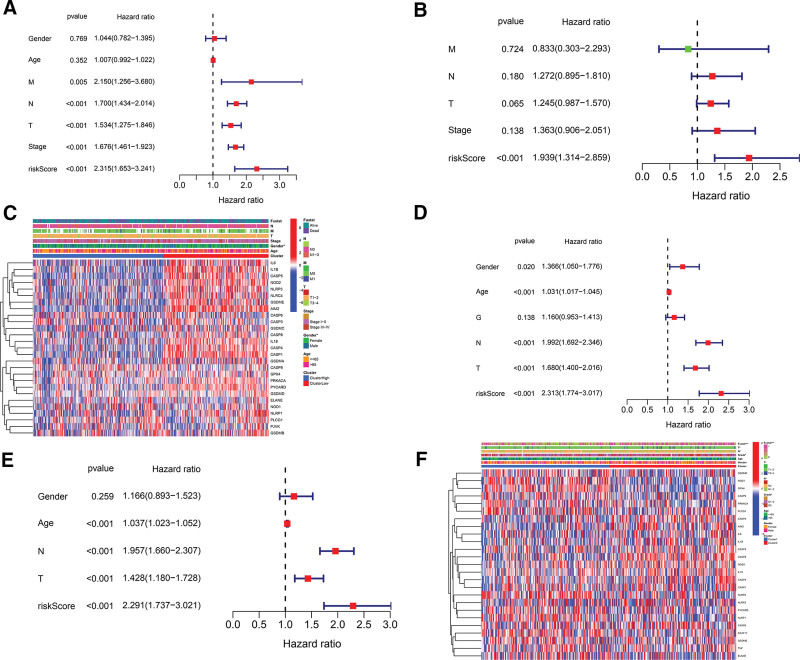
Univariate and multivariate Cox regression analyses for risk score. (A) Univariate Cox regression analysis and (B) multivariate Cox regression analysis verifying independent prognostic value of risk score for overall mortality in the TCGA cohort. (C) Heat map (blue: low expression; red: high expression) depicting correlation of gene and clinicopathologic features to mortality risk in the TCGA cohort. (D) Univariate Cox regression analysis and (E) multivariate Cox regression analysis confirming independent prognostic value of risk score for overall mortality in the GEO cohort. (F) Heat map (blue: low expression; red: high expression) for the association of gene and clinicopathologic features with mortality risk in the GEO cohort. G = grade for degree of tumor differentiation, GEO = Gene Expression Omnibus, M = stage for metastasis, N = stage for regional lymph node, T = stage for tumor, TCGA = The Cancer Genome Atlas.

The risk scores together with clinicopathological features (age, gender, grade, stage, M, N, and T) in the TCGA and GEO cohorts were submitted to univariate and multivariate Cox regression analysis. Univariate Cox regression analysis demonstrated that the risk score was associated with overall mortality of LUAD patients in both the TCGA cohort (hazard ratio [HR], 2.315; 95% confidence interval [CI], 1.653–3.241; *P* = 1.02 × 10^−6^) and the GEO cohort (HR, 2.313; 95% CI, 1.774–3.017; *P* = 6.11 × 10^−10^; Table 4; Fig. 6A and 6D). Multivariate analysis also displayed that the risk score was an independent risk of overall mortality for LUAD patients in the TCGA cohort (HR, 1.939; 95% CI, 1.314–2.859; *P* = 8.43 × 10^−4^) and the GEO cohort (HR, 2.291; 95% CI, 1.737–3.0201; *P* = 4.34 × 10^−9^; Fig. 6B and 6E) after adjusting for other confounding factors. In addition, we produced a heat map incorporating gene expression and clinicopathological traits for the TCGA and GEO cohorts individually (Table 4; Fig. 6C and 6F).

**Table 4:** Univariate and multivariate Cox analysis of risk score in conjunction with clinicopathological features.

Cohort	Univariate Cox regression					Multivariate Cox regression				
	Variable	HR	95% CI		*P* value	Variable	HR	95% CI		*P* value
			LL	UL				LL	UL	
TCGA	Gender	1.044	0.782	1.395	.769	Gender	-	-	-	-
	Age	1.007	0.992	1.022	.352	Age	-	-	-	-
	M	2.150	1.256	3.680	.005	M	0.833	0.303	2.293	0.724
	N	1.700	1.434	2.014	9.02 × 10^−10^	N	1.272	0.895	1.810	0.180
	T	1.534	1.275	1.846	5.78 × 10^−6^	T	1.245	0.987	1.570	0.065
	Stage	1.676	1.461	1.923	1.84 × 10^−13^	Stage	1.363	0.906	2.051	0.138
	Risk score	2.315	1.653	3.241	1.02 × 10^−6^	Risk score	1.939	1.314	2.859	0.000843
GEO	Gender	1.366	1.050	1.776	.020	Gender	1.166	0.893	1.523	0.259
	Age	1.031	1.017	1.045	1.26 × 10^−5^	Age	1.037	1.023	1.052	2.77 × 10^−7^
	N	1.992	1.692	2.346	1.38 × 10^−16^	N	1.957	1.660	2.307	1.25 × 10^−15^
	T	1.680	1.400	2.016	2.47 × 10^−8^	T	1.428	1.180	1.728	0.000249
	Grade	1.160	0.953	1.413	.138	Grade	-	-	-	-
	Risk score	2.313	1.774	3.017	6.11 × 10^−10^	Risk score	2.291	1.737	3.021	4.34 × 10^−9^

CI = confidence interval, Coef. = coefficient, GEO = Gene Expression Omnibus, HR = hazard ratio, LL = lower limit, M = metastasis, N = lymphoid node, T = tumor, TCGA = The Cancer Genome Atlas, UL = upper limit.

### 3.7. Cross-validation of prognostic signature

#### 3.7.1. Validation by the GEO cohort.

For validation of the 3-gene signature by the GEO cohort, the data for gene expression and clinicopathological information of 443 samples in GSE68454 series were used. Unexpectedly, *NLRC4*, a constituent gene within the 3-gene prognostic signature, is not available in the GSE68454 series. Consequently, the validation of the 3-gene signature by the GEO cohort could not be carried out.

#### 3.7.2. Validation by the TCGA cohort.

**Figure 7.**
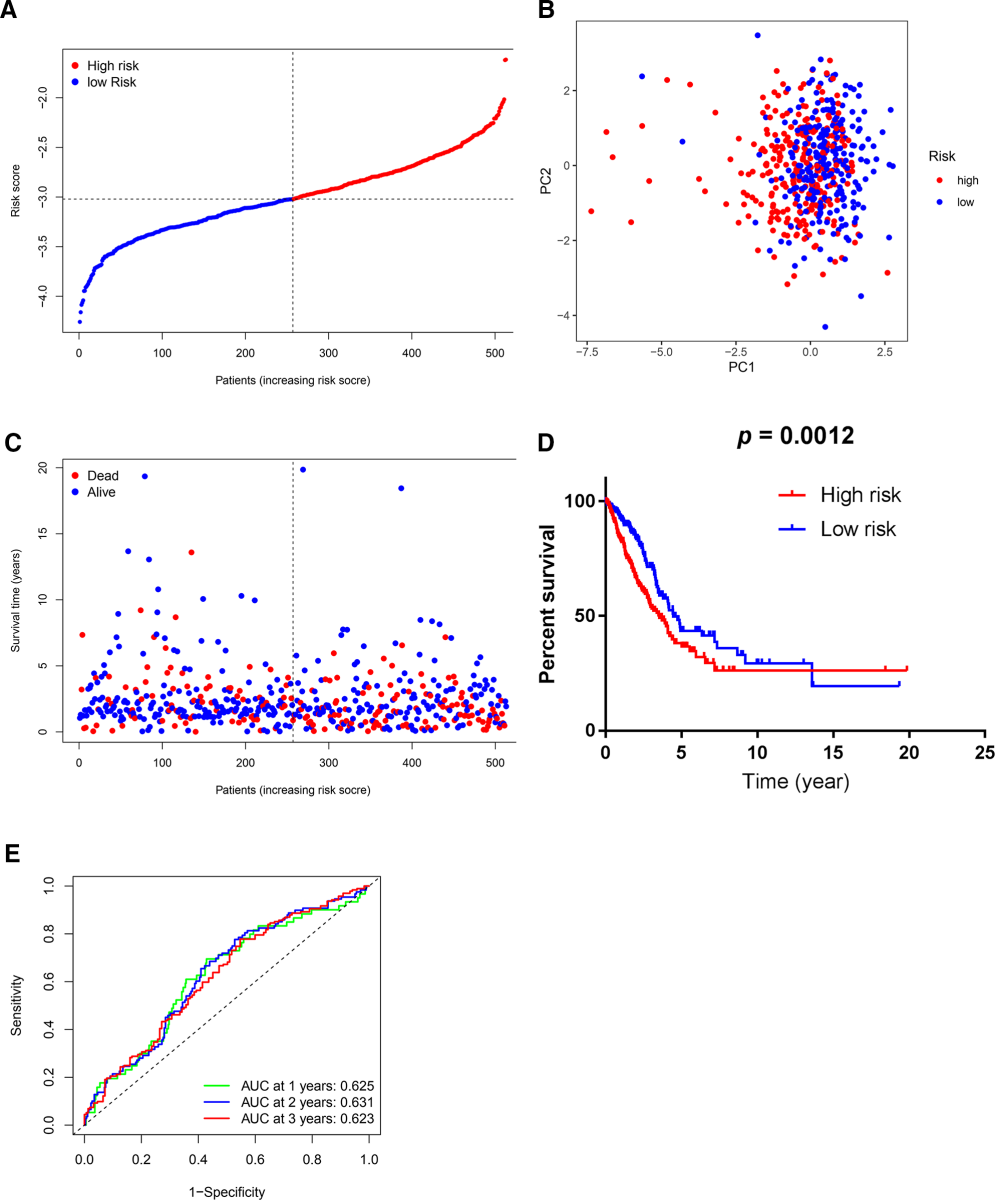
Validation of 7-gene prognostic signature by the TCGA cohort. (A) Distribution of the number of patients in low- and high-risk groups based on median value of risk scores. (B) Principal component analysis plot based on risk scores, separating LUAD patients into 2 clusters. (C) Survival status for each patient in the TCGA cohort (low-risk group: on the left side of the dotted line; high-risk population: on the right side of the dotted line; orange: higher risk score; blue: lower risk score). (D) Kaplan-Meier survival curves for OS of LUAD patients in high- and low-risk groups, indicating a lower OS rate in the high-risk group than in the low-risk group. (E) Receiver operating characteristic curves of predictive performance of prognostic signature for LUAD patients. LUAD = lung adenocarcinoma, OS = overall survival, TCGA = The Cancer Genome Atlas.

**Figure 8.**
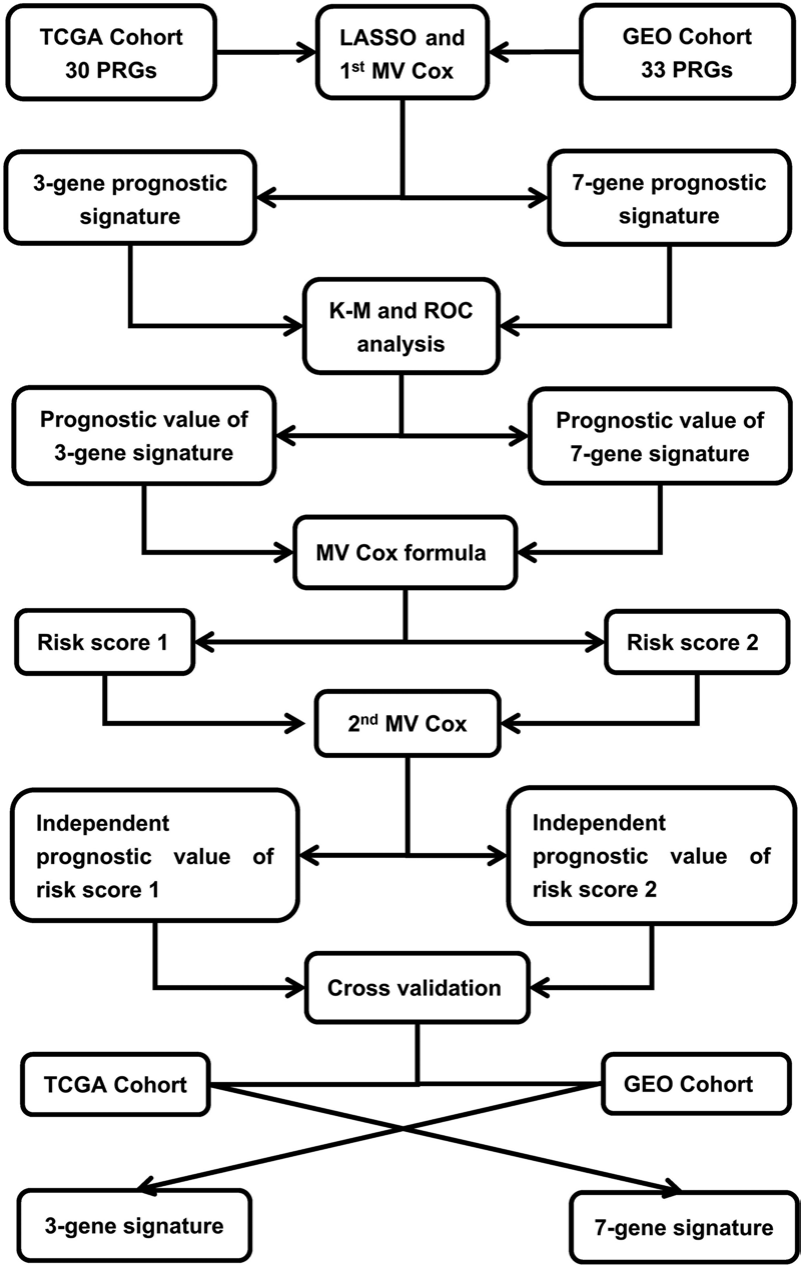
Flowchart delineating procedures for development of prognostic signatures and evaluation of independent prognostic value of risk scores in predicting overall mortality among LUAD patients. Three-gene prognostic signature denotes one consisting of *PYCARD*, *NLRP1*, and *NLRC4*. Seven-gene prognostic signature signifies another composed of *SCAF11*, *NOD1*, *NLRP2*, *NLRP1*, *GPX4*, *CASP8*, and *AIM2*. Risk score 1 is determined with regression coefficients and gene expression levels in the TCGA cohort using a MV Cox regression formula. Risk score 2 is calculated with regression coefficients and gene expression levels in the GEO cohort using another MV Cox regression formula. GEO = Gene Expression Omnibus, K-M = Kaplan-Meier, LASSO = least absolute shrinkage and selection operator, MV Cox = multivariate Cox regression, PRG = pyroptosis-related gene, ROC = receiver operating characteristic, TCGA = The Cancer Genome Atlas.

Based on the median value of risk scores that were generated by the 7-gene signature from the GEO cohort, 257 samples from the TCGA cohort were arranged into the low-risk group, while the other 256 patients were cataloged into the high-risk group (Fig. 7A). The PCA demonstrated suitable segregation between the 2 risk groups (Fig. 7B). As Fig. 7C manifests, the patients in the high-risk group suffer more deaths and have a shorter survival time than those in the low-risk group. As the Kaplan-Meier plots in Fig. 7D indicate, the patients in the high-risk group have a lower 5-year survival rate than patients in the low-risk group. The ROC curve analysis exhibits that the risk score yielded by the GEO cohort has a moderate predictive efficacy in TCGA (AUC, 0.625 for 1-year, 0.631 for 2-year, and 0.623 for 3-year survival; Fig. 7E). This validation by the TCGA cohort verified the prognostic value of the 7-gene signature originated from the GEO cohort for predicting LUAD outcome. The whole procedures for the development and validation of prognostic signatures are illustrated in a flowchart (Fig. 8).

### 3.8. Correlation of immune activity to mortality risk

**Figure 9.**
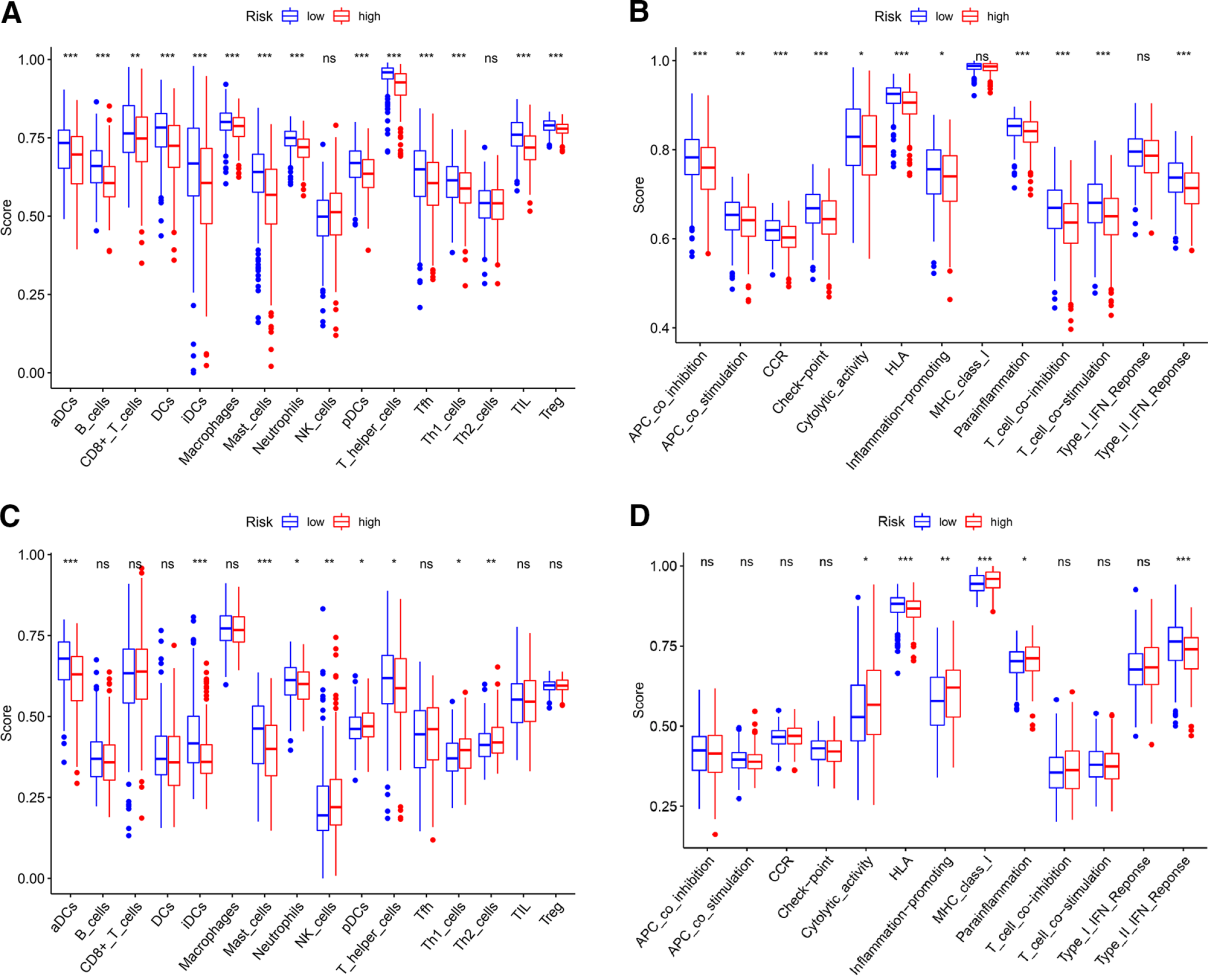
Box plots for comparing single-sample gene set enrichment analysis scores for 16 types of immune cells and 13 immune pathways between the low- and high-risk groups in the The Cancer Genome Atlas (A and B) and Gene Expression Omnibus cohorts (C and D). Considerably lower levels of infiltration of aDCs, B cells, iDCs, mast cells, and neutrophils, together with lower activity of type II IFN response and HLA, are observed in the high-risk group than in the low-risk group. *P* values were shown as ns for *P* > 0.05, * for *P* < 0.05, ** for *P* < 0.01, and *** for *P* < 0.001. ns = nonsignificant. aDC = activated dendritic cell, APC = antigen presenting cell, CCR = cytokine cytokine receptor, DC = dendritic cell, HLA = human leukocyte antigen, iDC = immature dendritic cell, IFN = interferon, MHC = major histocompatibility complex, NK = natural killer, pDC = plasmacytoid dendritic cell, Tfh = T follicular helper, Th = T helper, TIL = tumour-infiltrating lymphocyte, Treg = regulatory T cell.

In the TCGA and GEO cohorts, consistent findings concerning ssGSEA were achieved that the high-risk group had pronouncedly lower levels of infiltration of such immune-related cells as activated dendritic cells (aDCs), B cells, immature dendritic cells (iDCs), mast cells, and neutrophils (all *P* < 0.05; Fig. 9A and 9C). Correspondingly, in the 2 cohorts, analogous results were noticed that high-risk group had considerably lower activity in immune-related pathway of type II interferon (IFN) response and human leukocyte antigen (HLA) (Fig. 9B and 9D). However, the 2 cohorts did not coincide well in regard to infiltration levels of the other 11 immune cells and activity of the other 11 immune-related pathways.

## 4. Discussion

### 4.1. Main findings

In the present study, we estimated the roles of 30 PRGs playing in the occurrence and prognosis of LUAD using integrative bioinformatic analyses. Our findings revealed that most of the 30 genes were differentially expressed between normal lung and tumor tissues. Subsequently, we established a prognostic signature by combing 3 PRGs with the TCGA cohort and another signature by integrating 7 genes with the GEO cohort. Afterward, we testified the prognostic value of the 2 signatures via cross-validation of the TCGA and GEO cohorts by each other and finally certificated the only one valid and robust signature consisting of 7 PRGs (*SCAF11*, *NOD1*, *NLRP2*, *NLRP1*, *GPX4*, *CASP8*, and *AIM2*) for predicting the prognosis of LUAD patients.

Also, we performed GO and KEGG analyses and speculated that PRGs might play their roles in the occurrence of LUAD via dysregulated cytokines (IL-1 and IL-1B), inflammasome complex, and endopeptidase activity, and disordered pathway of NOD-like receptor signaling, cytosolic DNA sensing, and tumor necrosis factor signaling. Additionally, our ssGSEA uncovered that the LUAD patients with higher mortality risk that was defined by median risk score had substantially lower levels of infiltration of immune cells such as aDCs, B cells, iDCs, mast cells, and neutrophils and lower activity in the pathway of type II IFN response and HLA. These findings implied that poor prognosis of LUAD patients could be involved in impaired activity of immune cells and pathways.

### 4.2. Association of PRGs with prognosis of LUAD

#### 4.2.1. Absent in melanoma 2.

AIM2, as a representative of the NOD-like receptor family inflammasomes,^[16–18]^ activates a network of caspases including caspase-1 and caspase-8 and promotes both pyroptotic and apoptotic cell death.^[19]^ Kong et al^[20]^ documented that the AIM2 inflammasome was overexpressed in non–small cell lung cancer. Consistently, we noted that AIMs was among the 29 DEGs between normal lung and tumor tissues and that AIM2 expression was upregulated in our pooled GTEx and TCGA data, supporting a correlation of high AIM2 expression to the occurrence of LUAD. Moreover, Colarusso et al^[16]^ declared that higher expression of AIM2 in LUAD patients was correlated to a higher HR of poor survival. In agreement with Colarusso et al’s findings, our multivariate regression results admitted independent association of higher AIM2 expression with higher overall mortality in LUAD patients.

#### 4.2.2. Cysteine-aspartic acid protease-8.

As described by Sagulenko et al^[19]^ in their work, caspase-1 drives rapid lysis of cells by pyroptosis and maturation of IL-1β and IL-18, while CASP8 represents the molecular switch that controls apoptosis, necroptosis, and pyroptosis.^[21]^ In cells where rapid pyroptosis is blocked, delayed inflammasome-dependent cell death still occurs due to both caspase-1–dependent and caspase-8–dependent apoptosis.^[19]^ However, there has been a lack of knowledge in existing studies dwelling on the mechanism responsible for the correlation of CASP8 to the development and prognosis of LUAD. Our finding showed that CASP8 was downregulated in tumor tissues and suggested its negative association with the occurrence of LUAD. Our finding is not in conformity with that in a study undertaken by Kutilin et al,^[22]^ who found that the proportion of gene copy numbers for CASP8 was not significantly different between tumor cells and normal lung cells. In this study, we identified CASP8 as an independent risk factor for overall mortality in LUAD patients, which is in concordance with Liu et al’s conclusion.^[2]^

#### 4.2.3. Glutathione peroxidase 4.

GPX4 functions as an antioxidant enzyme and key enzyme that protects cells from lipid peroxidation by way of metabolizing reactive oxygen species and reactive carbonyl species.^[23]^ Romanowska et al^[24]^ documented in their work that GPX4 was upregulated in LUAD cell lines. In line with their evidence, our results from pooled GTEx and TCGA cohort indicated upregulated GPX4 expression in LUAD tissues relative to normal lung tissues. Another analysis of public databases by Lei et al^[25]^ disclosed that in patients undergoing chemotherapy, those with higher expression of GPX4 had a lower probability of survival. Lei et al’s result is not in conformity with ours that GPX4 is significantly reversely associated with the overall mortality of LUAD patients by multivariate Cox regression analysis. The mechanism where there exists a relationship between GPEx expression and prognosis in LUAD patients has not been fully addressed yet.

#### 4.2.4. NLRP1 and NLRP3.

NLR family, NLRP1 and NLRP3, are the 2 best-characterized inflammasome members.^[26]^ NLRP1, the first NLR protein, serves as an inflammasome sensor to induce cytokine maturation and pyroptosis by mediating the activation of caspase-1.^[27,28]^ In response to activation by many different stimuli, increased NLRP3 protein expression and inflammasome assembly lead to caspase-1–mediated maturation and release of IL-1β, which triggers inflammation and pyroptosis.^[29]^ Shen et al^[30]^ performed a bioinformatics analysis of LUAD data that were downloaded from TCGA and GEO and noticed that NLRP1 expression in LUAD tissues was considerably lower than that in normal lung tissues. Homogeneously, our findings evidenced that NLRP1 and NLRP3 were significantly downregulated in tumor compared to normal lung tissues. Furthermore, they affirmed that reduced NLRP1 expression level was an independent prognostic factor of LUAD patients by multivariate Cox regression analysis.^[30]^ In symphony with Shen et al’s findings, ours revealed that lower gene expression of NLRP1 is independently associated with poor prognosis in patients in the TCGA LUAD cohort.

#### 4.2.5. NLRP2, NOD1, and SCAF11.

*NLRP2*, *NOD1*, and *SCAF11* were all DEGs and components in our prognostic signatures with the GEO cohort. However, less has been known with respect to the mechanism by which the 3 genes play their roles in the development and prognosis of LUAD.

### 4.3. Qualified prognostic signature

In this study, we screened candidate genes within 30 PRGs and identified and validated a prognostic PRG signature integrating 7 genes for predicting the probability of mortality in LUAD patients. Constituents in our 7-gene signature (*SCAF11*, *NOD1*, *NLRP2*, *NLRP1*, *GPX4*, *CASP8*, and *AIM2*) are considerably different from Lin et al’s (*NLRP7*, *NLRP1*, *NLRP2*, *NOD1*, and *CASP6*)^[9]^ in terms of composition, which is likely to be attributed to the difference in data source.

### 4.4. Clinical implications

In this study, we preliminarily investigated prognostic value of PRGs and provided theoretical support for future research. Moreover, our prognostic signature is expected to be applied to clinical practice, which means that, first, it may be a promising indicator for prognosis of LUAD patients; second, it helps clinicians identify LUAD patients at high risk of mortality; and third and more importantly, it provides a potential therapeutic strategy for LUAD in the future.

### 4.5. Superiority

Above all, we conducted cross-validation of our 2 prognostic signatures by using the GEO and TCGA cohorts alternately, which strengthened the robustness of our conclusion in terms of prognostic value of our 7-gene prognostic signature. What’s more, we used data from pooled GTEx and TCGA cohorts for comparisons of gene expression levels and for the identification of the hub gene, which facilitated minimizing the bias that is attributed to a relatively lack and imbalance of data from normal lung tissues in the TCGA cohort.

### 4.6. Limitations

We confess there exist 2 inherent limitations in our study. First and foremost, our study is merely a bioinformatics analysis without laboratory validation of differential expression and clinical verification of prognostic performance for our 7-gene signature in our own data sets of patients. Secondarily, we fail to use the GEO LUAD cohort to validate prognostic signature that is constructed by the TCGA LUAD cohort, since the data for NLRC3 mRNA expression and corresponding survival status are not available in the GEO LUAD cohort. Pooling ≥2 data series from GEO archives might be hopeful to offset the limitation.

## 5. Conclusion

Taken together, we have constructed and cross-validated a prognostic signature incorporating 7 PRGs (*SCAF11*, *NOD1*, *NLRP2*, *NLRP1*, *GPX4*, *CASP8*, and *AIM2*) with the GEO and TCGA cohorts for predicting prognosis for LUAD patients. Furthermore, our study confirms that the risk score generated from the 7-gene prognostic signature is an independent risk factor for poor prognosis of LUAD patients.

## Acknowledgments

The authors would like to acknowledge the TCGA, GTEx, and GEO network for providing data.

## Author contributions

Conceptualization: Yingchun Jiang.

Data curation: Zhaohui Qu, Jing Ge, Chunxia Zhang, and Chunfang Li.

Formal analysis: Wei Zhang.

Investigation: Shiqian Wan.

Methodology: Wei Zhang.

Resources: Zhaohui Qu, Jing Ge, Chunxia Zhang, and Chunfang Li.

Software: Wei Zhang.

Supervision: Yingchun Jiang.

Validation: Shiqian Wan.

Visualization: Wei Zhang.

Writing – original draft: Wei Zhang.

Writing – review & editing: Shiqian Wan and Yingchun Jiang.
